# Impact of the 2024 Organ Procurement and Transplantation Network Kidney Donor Profile Index Rescoring on Donor Kidney Utilization and Allocation out of Sequence: A one‐Year Analysis

**DOI:** 10.1111/ctr.70604

**Published:** 2026-06-23

**Authors:** Shengliang He, Christie P. Thomas, Alan I. Reed

**Affiliations:** ^1^ Department of Surgery Division of Transplant & Hepatobiliary Surgery Organ Transplant Center University of Iowa Hospital & Clinics Iowa City Iowa USA; ^2^ Department of Internal Medicine University of Iowa Carver College of Medicine Iowa City Iowa USA; ^3^ VA Medical Center Iowa City Iowa USA

**Keywords:** allocation out‐of‐sequence (AOOS), donor race, Hepatitis C virus (HCV), Kidney Donor Profile Index (KDPI), kidney utilization

## Abstract

**Background:**

In October 2024, the Organ Procurement and Transplantation Network (OPTN) removed hepatitis C virus (HCV) positivity and African American/Black (AA/B) donor race from the Kidney Donor Profile Index (KDPI) calculation. This study evaluates the early impact of this policy change on kidney utilization during its first year.

**Methods:**

We performed a retrospective analysis of OPTN data for organ procurements and adult deceased‐donor kidney transplants between November 2023 and October 2025. Donors were stratified by HCV status and race. Kidney utilization and allocation out‐of‐sequence (AOOS) were compared between pre‐policy (November 2023–October 2024) and post‐policy (November 2024–October 2025) periods.

**Results:**

The policy revision was associated with redistribution of HCV+ and AA/B donor kidneys toward lower KDPI category, with increases of 989% and 144%, respectively, in the 0–20% KDPI range. Overall utilization increased among AA/B (66.9% to 71.5%), non‐AA/B (70.1% to 72.1%), HCV− (69.6% to 72.0%), and HCV+ donors (70.3% to 72.5%). However, utilization within individual KDPI categories declined for AA/B and HCV+ donors across most strata, except for the 21–34% category among HCV+ donors. AOOS decreased from 20.2% to 16.8% (absolute reduction 3.4%; 95% confidence interval, −4.1% to −2.6%).

**Conclusion:**

The policy was associated with increased overall utilization across donor groups. However, gains among AA/B and HCV+ donors appear primarily driven by redistribution into different allocation sequences rather than improvement in within‐category utilization. Reduced AOOS suggests improved allocation efficiency, though findings may be influenced by concurrent regulatory changes.

**Abbreviations**
AA/B: African American/Black
AOOS: Allocation out‐of‐sequence
CI: Confidence intervals
HCV ‐: HCV negative
HCV +: HCV positive
HCV: Hepatitis C virus
HRSA: Health Resources and Services Administration
KDPI: Kidney Donor Profile Index
KDRI: Kidney Donor Risk Index
NAT: Nucleic acid testing
OPTN: Organ Procurement and Transplantation Network
STAR: Standard Analysis and Research

## 1 Introduction

On October 31, 2024, the Organ Procurement and Transplantation Network (OPTN) implemented a policy removing hepatitis C virus (HCV) positivity and donor race from the Kidney Donor Profile Index (KDPI) calculation [1]. This revision was intended to improve the accuracy of graft failure risk estimation by eliminating variables that no longer reflect contemporary clinical outcomes and to promote more racially equitable distribution of donor organs [2]. Race has been recognized as an imprecise proxy for genetic variation, and advances in antiviral therapy have resulted in post‐transplant outcomes for HCV positive deceased donor kidneys that are comparable to those of HCV negative donors [3, 4].

KDPI was used in 2014 as part of the implementation of the Kidney Allocation System to align expected graft longevity with recipient survival [5]. KDPI is derived from the Kidney Donor Risk Index (KDRI), that was originally calculated based on ten donor‐specific factors: age, height, weight, history of diabetes and hypertension, serum creatinine, hepatitis C status (serology or nucleic acid testing, NAT), ethnicity, cause of death, and donation after cardiac death. The original KDRI model was derived from transplants performed between 1995 and 2005 [6]. The updated model used in the 2024 policy change was refit using 2018–2021 data and demonstrated that removal of donor Black race and HCV status resulted in more equitable KDPI distributions with minimal projected impact on overall nonuse [7].

The revised KDPI was anticipated to enhance equity and transparency within the national organ allocation system and may reduce nonuse of deceased donor kidneys. However, the real‐world effect of this policy on kidney utilization has not been evaluated. We therefore examined early changes in kidney utilization during the first year following implementation of the revised KDPI calculation.

## 2 Methods

### 2.1 Study Population

This study utilized data from the OPTN Standard Analysis and Research (STAR) file maintained by the United Network for Organ Sharing. The OPTN STAR file contains data submitted by members on all donors, waitlisted candidates, and transplant recipients in the United States (US). The Health Resources and Services Administration (HRSA) oversees the activities of OPTN and its contractees. This retrospective cohort study included all registered kidney transplants recorded in the OPTN STAR files between November 1, 2023, and October 31, 2025. The study period was divided into a pre‐policy era (November 1, 2023–October 31, 2024) and a post‐policy era (November 1, 2024–October 31, 2025), corresponding to implementation of the allocation policy change. The study was exempted from review by the University of Iowa Institutional Review Board (Project 202410636).

In this study we examined the outcome of procured deceased donor kidneys from adult (≥18 years) donors. Donor kidneys are categorized into four allocation sequences based on KDPI values: Sequence A (KDPI 0%–20%), Sequence B (KDPI 21%–34%), Sequence C (KDPI 35%–85%), and Sequence D (KDPI 86%–100%), each with its own allocation priorities [8]. Donors were further categorized by HCV status as positive or negative and by donor race as African American/Black (AA/B) or non‐AA/B. In this study, an HCV+ donor was defined as a subject with either a positive HCV NAT result or positive HCV antibody status. An HCV—donor was defined as a subject with a negative HCV NAT and negative HCV antibody status. For utilization analyses, all kidneys procured during the study period were included regardless of whether transplantation occurred; donors with missing HCV status were excluded from HCV‐related analyses. Allocation out‐of‐sequence (AOOS) is when an organ is offered or accepted or transplanted into a transplant candidate or potential transplant recipient that deviates from the match sequence and is not consistent with OPTN policy. AOOS is identified through the use of one or more of the following organ offer bypass codes: 861: Operational—Organ Procurement Organization; 862: Donor medical urgency; 863: Offer not made due to expedited placement attempt; 887: Not Offered—expedited placement; 799: Other (specify).

### 2.2 Outcome Ascertainment

The primary outcome was kidney utilization, defined as the proportion of procured kidneys that were transplanted within each KDPI category and stratified by HCV status and donor race. Secondary outcomes included KDPI distribution among AA/B and HCV+ donors, as well as the proportion of AOOS percentage, defined as the proportion of transplanted kidneys allocated out of sequence among all transplanted kidneys within each KDPI sequence. Outcomes were assessed separately in the pre‐policy and post‐policy eras.

### 2.3 Statistical Analysis

Utilization and AOOS rates were calculated as proportions within each sequence. The utilization rate was defined as the proportion of transplanted kidneys among procured kidneys, whereas the AOOS rate was defined as the proportion of transplanted kidneys allocated out of sequence among all transplanted kidneys. Absolute differences between post‐policy and pre‐policy periods were expressed as percentage‐point changes (Δ = post minus pre). Effect estimates are reported as absolute percentage‐point differences with corresponding 95% confidence intervals (CIs). Statistical significance was determined by 95% CIs that did not include 0. All tests were two‐sided, and analyses were conducted using Python version 3.10 (Python Software Foundation, Wilmington, DE, USA).

## 3 Results

### 3.1 Distribution of AA/B and HCV + Donors, Stratified by KDPI Category

Table 1 demonstrated that overall proportion of AA/B donors remained similar between the pre‐ and post‐policy periods (14.2% vs 13.5%). However, their distribution across KDPI categories shifted substantially. The proportion of AA/B donors increased by 144.3% in the lowest KDPI category (0%–20%), rising from 6.9% to 16.9% (Δ +10.0%, 95% CI 8.6 to 11.4), with 711 kidneys procured post‐policy compared to 300 pre‐policy. In contrast, it decreased by 45.9% in the highest KDPI category (86%–100%), from 22.9% to 12.4% (Δ −10.6%, 95% CI −12.0 to −9.1), with 770 kidneys procured post‐policy versus 1519 pre‐policy. Minimal changes were observed in the 21%–35% and 36%–85% KDPI categories.

**TABLE 1:** Percentage of African American/Black and HCV‐positive donors, stratified by KDPI category.

	African American/Black			HCV positive		
KDPI	Pre‐policy	Post‐policy	Δ (95% CI)	Pre‐policy	Post‐policy	Δ (95% CI)
0%–20%	6.90% (300/4346)	16.86% (711/4217)	+9.96 (8.57 to 11.35)	0.37% (16/4346)	4.03% (170/4217)	+3.66 (3.03 to 4.29)
21%–34%	13.40% (478/3567)	13.82% (452/3271)	+0.42 (−1.16 to 2.00)	2.47% (88/3565)	4.53% (148/3267)	+2.06 (1.16 to 2.96)
35%–85%	13.48% (1943/15402)	12.88% (1935/15027)	−0.60 (−1.37 to 0.17)	5.33% (820/15392)	4.23% (636/15021)	−1.10 (−1.59 to −0.61)
86%–100%	22.94% (1519/6622)	12.36% (770/6231)	−10.58 (−12.02 to −9.14)	5.91% (391/6622)	2.60% (162/6229)	−3.31 (−4.02 to −2.60)
Total	14.16% (4240/29937)	13.45% (3868/28746)	−0.71 (−1.26 to −0.16)	4.40% (1315/29925)	3.88% (1116/28734)	−0.52 (−0.86 to −0.18)

*Note:* Pre‐policy refers to November 1, 2023, to October 31, 2024; post‐policy refers to November 1, 2024, to October 31, 2025. Values are presented as percentages (n/N), where n denotes African American/Black or HCV‐positive kidneys and N denotes the total number of kidneys procured within each KDPI category. Δ represents absolute percentage‐point change (post‐policy minus pre‐policy).

Abbreviations: CI, confidence interval; HCV, hepatitis C virus; KDPI, Kidney Donor Profile Index.

In contrast, HCV+ donors comprised a small proportion of the overall donor population but demonstrated significant shifts across multiple KDPI categories. The proportion of HCV+ donors increased 989% in the lowest KDPI category (0%–20%; 0.4% to 4.0%) and 83.4% in the 21%–34% category (2.5% to 4.5%), while decreasing in the 35%–85% (5.3% to 4.2%) and 86%–100% categories (5.9% to 2.6%). Despite these shifts, the overall proportion of HCV+ donors declined slightly over time (4.4% to 3.9%).

### 3.2 Kidney Utilization Stratified by Race

Table 2 summarizes kidney utilization before and after policy implementation stratified by KDPI category and donor race. There was an overall decrease in both the number of organs procured (29 937 vs. 28 746) and the number of kidney transplants performed (20 854 vs. 20 699) in the pre‐ versus post‐policy change periods. Overall utilization increased for both AA/B and non‐AA/B donors in the post‐policy era. Utilization increased from 66.9% pre‐policy to 71.5% post‐policy from AA/B donors, corresponding to an absolute increase of 4.6% (95% CI, 2.5% to 6.6%). Utilization increased from 70.1% to 72.1% from non‐AA/B donors, representing an absolute increase of 2.0% (95% CI, 1.2% to 2.8%).

**TABLE 2:** Kidney utilization rate pre and post policy implementation, stratified by KDPI category and race.

	African American/Black			Non‐ African American/Black		
KDPI	Pre‐policy	Post‐policy	Δ (95% CI)	Pre‐policy	Post‐policy	Δ (95% CI)
0%–20%	98.7% (296/300)	95.6% (680/711)	−3.1 (−5.01 to −1.04)	96.8% (3916/4046)	97.0% (3400/3506)	+0.2 (−0.60 to 0.97)
21%–34%	93.3% (446/478)	91.8% (415/452)	−1.5 (−4.87 to 1.89)	92.7% (2862/3089)	95.3% (2686/2819)	+2.6 (1.42 to 3.84)
35%–85%	78.9% (1534/1943)	72.6% (1405/1935)	−6.3 (−9.03 to −3.65)	73.1% (9842/13459)	76.6% (10031/13092)	+3.5 (2.45 to 4.54)
86%‐100%	37.0% (562/1519)	34.4% (265/770)	−2.6 (−6.72 to 1.56)	27.4% (1396/5103)	33.3% (1817/5461)	+5.9 (4.17 to 7.66)
Total	66.9% (2838/4240)	71.5% (2765/3868)	+4.6 (2.54 to 6.56)	70.1% (18016/25697)	72.1% (17934/24878)	+2.0 (1.19 to 2.77)

*Note:* Pre‐policy refers to November 1, 2023–October 31, 2024; post‐policy refers to November 1, 2024–October 31, 2025. Values are percentage (n/N). Δ represents absolute percentage‐point change (post‐policy minus pre‐policy).

Abbreviations: CI, confidence interval; KDPI, Kidney Donor Profile Index.

Utilization in individual KDPI categories differed significantly by donor race. Among AA/B donors, the largest shift occurred in the 0%–20% KDPI category, with 680 kidneys transplanted post‐policy versus 296 pre‐policy. However, by proportion, utilization declined by 3.1% (98.7% vs. 95.6%; 95% CI, −5.0% to −1.0%). A similar pattern was observed in the 35%–85% KDPI category, with 1534 kidneys transplanted pre‐policy versus 1405 post‐policy, and utilization declining by 6.3% (78.9% vs. 72.6%; 95% CI, −9.0% to −3.7%). Utilization among AA/B donors also decreased in both the 21%–34% and 86%–100% KDPI categories following the policy change; however, these changes were not statistically significant.

In contrast, among non‐ AA/B donors, utilization increased significantly in multiple KDPI categories. Utilization increased by 2.6% (95% CI, 1.4% to 3.8%) in the KDPI 21%–34% category, by 3.5% (95% CI, 2.5% to 4.5%) in the KDPI 35%–85% category, and by 5.9% (95% CI, 4.2% to 7.7%) in the KDPI 86%–100% category. Utilization in the KDPI 0%–20% category showed a slight increase from 96.8% to 97.0%; however, this change was not statistically significant (Δ +0.2%, 95% CI −0.60 to 0.97).

### 3.3 Kidney Utilization Stratified by HCV Status

Table 3 summarizes kidney utilization before and after policy implementation stratified by KDPI category and donor HCV status. Overall utilization of HCV—kidneys increased from 69.6% pre‐policy to 72.0% post‐policy, corresponding to an absolute increase of 2.4% (95% CI, 1.6% to 3.1%). Overall utilization of HCV+ kidneys increased from 70.3% to 72.5%, however the change was not statistically significant (Δ +2.2, 95% CI: −1.38 to 5.83).

**TABLE 3:** Kidney utilization rate pre and post policy implementation, stratified by KDPI category and HCV status.

	HCV positive			HCV negative		
KDPI	Pre‐policy	Post‐policy	Δ (95% CI)	Pre‐policy	Post‐policy	Δ (95% CI)
0%–20%	100.0% (16/16)	97.1% (165/170)	−2.9 (−5.48 to −0.40)	96.9% (4196/4330)	96.7% (3915/4047)	−0.2 (−0.92 to 0.59)
21%–34%	95.5% (84/88)	95.9% (142/148)	+0.4 (−4.90 to 5.88)	92.7% (3222/3477)	94.8% (2957/3119)	+2.1 (0.97 to 3.30)
35%–85%	83.5% (685/820)	70.6% (449/636)	−12.9 (−17.30 to −8.58)	73.4% (10687/14572)	76.4% (10983/14385)	+3.0 (2.01 to 4.01)
86%–100%	35.5% (139/391)	32.7% (53/162)	−2.8 (−11.48 to 5.81)	29.2% (1819/6231)	33.5% (2029/6067)	+4.3 (2.61 to 5.89)
Total	70.3% (924/1315)	72.5% (809/1116)	+2.2 (−1.38 to 5.83)	69.6% (19924/28610)	72.0% (19884/27618)	+2.4 (1.61 to 3.11)

*Note:* Pre‐policy refers to November 1, 2023–October 31, 2024; post‐policy refers to November 1, 2024–October 31, 2025. Values are percentage (n/N), where n denotes transplanted kidneys and N denotes procured kidneys. Δ represents absolute percentage‐point change (post‐policy minus pre‐policy).

Abbreviations: CI, confidence interval; HCV, hepatitis C virus; KDPI, Kidney Donor Profile Index.

Among HCV + kidneys, utilization declined significantly in the KDPI 35%–85% category by 12.9% (95% CI, −17.3% to −8.6%) and in the KDPI 0%–20% category by 2.9% (95% CI, −5.5% to −0.4%). No significant changes were observed in the KDPI 21%–34% (95.5% to 95.9%, Δ +0.4%, 95% CI: −4.90 to 5.88) or 86%–100% (35.5% to 32.7%, Δ −2.8%, 95% CI:−11.48 to 5.81) categories.

In contrast, utilization of HCV—kidneys increased significantly in multiple KDPI categories. Utilization increased by 3.0% (95% CI, 2.0% to 4.0%) in the KDPI 35%–85% category, by 2.1% (95% CI, 1.0% to 3.3%) in the KDPI 21%–34% category, and by 4.3% (95% CI, 2.6% to 5.9%) in the KDPI 86%–100% category. No significant change was observed in the KDPI 0%–20% category.

### 3.4 AOOS Kidney Utilization

Table 4 summarizes AOOS before and after policy implementation stratified by KDPI category. Overall AOOS declined from 20.2% pre‐policy to 16.8% post‐policy, corresponding to an absolute reduction of 3.4% (95% CI, −4.1% to −2.6%).

**TABLE 4:** Allocation out‐of‐sequence before and after policy implementation, stratified by KDPI category.

KDPI	Pre‐policy	Post‐policy	Δ (95% CI)
0%–20%	8.0 (359/4492)	5.6 (256/4595)	−2.4 (−3.5 to −1.4)
21%–34%	13.5 (495/3668)	9.5 (322/3372)	−3.9 (−5.4 to −2.5)
35%–85%	24.8 (3030/12221)	20.4 (2524/12388)	−4.4 (−5.5 to −3.4)
86%–100%	29.6 (705/2381)	30.7 (705/2294)	1.1 (−1.5 to 3.8)
Total	20.2 (4589/22762)	16.8 (3807/22649)	−3.4 (−4.1 to −2.6)

*Note:* Pre‐policy refers to November 1, 2023–October 31, 2024; post‐policy refers to November 1, 2024–October 31, 2025. Values are percentage (n/N). Δ represents absolute percentage‐point change (post‐policy minus pre‐policy).

Abbreviations: CI, confidence interval; KDPI, kidney donor profile index.

Significant reductions in AOOS were observed across multiple KDPI categories. AOOS decreased by 4.4% (95% CI, −5.5% to −3.4%) in the KDPI 35%–85% category, by 3.9% (95% CI, −5.4% to −2.5%) in the KDPI 21–34% category, and by 2.4% (95% CI, −3.5% to −1.4%) in the KDPI 0–20% category. No significant change was observed in the KDPI 86%–100% category.

## 4 Discussion

Analyses of the data from the first 12 months following the 2024 OPTN policy update demonstrate increased utilization of donor kidneys despite an overall decline in the total number of transplants performed during the same period. Utilization rates increased across donor groups regardless of race or HCV status; increases in all donor categories reached statistical significance except for HCV+ kidneys, where the increase was not statistically significant. The pattern of utilization among HCV+ kidneys following the policy revision was not entirely unexpected. Since the publication of pivotal pilot studies in 2017 [3], the use of HCV viremic donor organs has become increasingly widespread. The transplant community has largely accepted the safety and efficacy of transplanting these organs into HCV—recipients in the era of direct‐acting antiviral therapy [9]. The previously inflated KDPI scoring system that incorporated HCV serostatus as a risk variable has long been recognized as a limitation [10, 11, 12]. Emerging evidence suggests that transplant surgeons may have already adjusted for inaccuracies in KDPI calculations when evaluating HCV‐viremic organs [13]. Notably, HCV + kidneys demonstrated relatively high utilization rates even prior to the 2024 policy change reflecting growing clinical confidence and established practice patterns.

Over the past two decades, multiple policy changes have been implemented to address systemic disparities in kidney transplantation [14]. These have included, in 2003, removal of allocation priority based on HLA‐B matching, in 2014, backdating of waiting time to the start of chronic dialysis, and permitting transplantation of blood type A2 and A2B deceased donor kidneys to B blood type transplant candidates (∼18%–20% of US African American/Black population vs ∼9%–10% in European ancestry [15]) and in 2023, rectification of lost waiting time from use of race‐inclusive estimated glomerular filtration rate equations for Black waitlisted candidates. The 2024 policy revision eliminating race from the KDPI calculation was initiated by the OPTN Minority Affairs Committee that asserted race is an imprecise proxy for human genetic variation, given that it is a social construct without consistent biological basis [16, 17].

Removal of race from the KDPI calculation was expected to improve scores for kidneys from Black donors, thereby altering allocation sequences for some of them and promoting a more racially equitable KDPI distribution [7]. Prior studies have demonstrated that lower KDPI scores are associated with greater organ acceptance and higher overall utilization rates [18].

Although an overall increase in utilization of kidneys from AA/B donors was observed following policy implementation, this increase appears to be largely attributable to redistribution of procured organs across allocation sequences. Both AA/B and HCV+ donor kidneys had a significantly lower proportion in high KDPI categories and a corresponding increase in lower KDPI categories. Despite categorizing more kidneys into the lower KDPI categories, especially into the 0%–20% category, utilization rates within individual allocation sequences declined following the policy change, suggesting that attributes associated with race and HCV status are still influencing utilization.

These findings raise the question of whether differential acceptance practices may persist despite modifications to allocation policy. The KDPI was developed to estimate graft longevity and align donor organ quality with recipient life expectancy to optimize allocation. However, the coefficient previously assigned to Black donor race (0.15) remained embedded in the updated risk modeling framework which can influence clinical interpretation and decision‐making. This observation suggests the possibility that transplant centers may have adjusted their acceptance behavior based on prior experience or entrenched perceptions, potentially attenuating the intended impact of the policy change on equitable organ utilization. However, it is also possible that the full impact of the policy change may take a few years to be reflected in kidney utilization rates.

Allocation out of sequence (AOOS) occurs when an organ is offered, accepted, and/or transplanted in a manner that deviates from the match run and is inconsistent with OPTN policy. Historically, AOOS was intended as a rare exception, used only when established allocation policies were insufficient to place a donated organ with an appropriate recipient. As recently as 2018, approximately 1 in 45 transplanted organs were allocated out of sequence. Beginning in 2019, the frequency of AOOS steadily increased, accelerating such that by 2024 nearly 1 in 5 transplanted organs were allocated outside the match sequence [19]. Several recent studies have examined potential drivers of this shift in practice [20, 21, 22]. Contributing factors included increased procurement of higher KDPI and donation after circulatory death organs with late allocation, misalignment between stated organ acceptance criteria and clinical practice, heightened time sensitivity for organ viability, and longer transport distances associated with the transition from allocation prioritized within donation service areas to allocation within concentric circles. The 2024 OPTN policy update was designed to improve equity and transparency in kidney allocation and to reduce organ nonuse, in part through revisions to risk assessment metrics. Under this framework, KDPI recalibration could plausibly affect AOOS through changes in perceived organ quality and allocation priority, thereby altering the likelihood that a kidney is placed within versus outside the standard sequence. A notable inflection point in AOOS utilization followed the August 2025 action by the HRSA and OPTN addressing AOOS practices [23]. A multidisciplinary workgroup was convened to establish standardized AOOS definitions through the OPTN, alongside efforts to enhance transparency via public reporting and a data dashboard to monitor AOOS trends. Although the 2024 policy change may have influenced AOOS trends, disentangling its independent effect from concurrent systemic and regulatory changes was beyond the scope of the present study.

Our study has several limitations. First, measures of donor quality have been evolving in recent years [24], and direct comparison of donor organ quality across eras is challenging because the prior and updated KDRI models differ substantially in the number of variables included (10 vs. 8). Second, their corresponding coefficient estimates differ, and the concordance of both models remains suboptimal. Third, the absence of offer‐level data precludes detailed assessment of decision‐making processes at the center level, and causality regarding organ utilization or discard cannot be inferred. Finally, contemporaneous policy changes in kidney allocation can confound the observed associations, particularly in analyses evaluating trends in AOOS. As such, the independent effect of the 2024 policy revision should be interpreted with caution.

## 5 Conclusion

An increase in the utilization of kidneys from AA/B donors was observed in the first 12 months following the policy change, whereas no comparable increase was noted among HCV+ donors. The observed gains among AA/B donors appear to be driven primarily by redistribution into lower KPDI allocation sequences rather than improved within‐category utilization, as utilization rates declined across most allocation sequences following policy implementation despite KDPI recalibration intended to prioritize both groups. Improved KDPI scores may have contributed to the observed reduction in out‐of‐sequence allocation as well, although the above finding can be confounded by other recent regulation changes. Ongoing evaluation is warranted to determine the long‐term impact of this policy change on equity, allocation efficiency, and organ utilization.

## Author Contributions

All the authors contributed to Concept/design, Data analysis/interpretation, Drafting article, Critical revision of article, Approval of article.

## Funding

The authors have nothing to report.

## Conflicts of Interest

Alan Reed is a member of the Board of Directors of the Organ Procurement and Transplantation Network (OPTN), and this manuscript includes an analysis of OPTN data for all adult deceased donor kidney transplants between November 1, 2023, and October 31, 2025, including a comparison of kidney utilization rates and allocation out‐of‐sequence across different periods.  The views, information, arguments, analyses, and opinions expressed in this manuscript are solely those of Alan Reed and the other authors in their individual capacities and do not represent the official position, policy, or endorsement of the OPTN, the U.S. Department of Health and Human Services, or any other public or private organization with which any of the authors are affiliated.  The OPTN has not reviewed or verified the analyses or conclusions presented in this manuscript.  Further, the use and inclusion of the OPTN's data does not imply the OPTN's endorsement of the authors' findings, methodology, or conclusions. Christie Thomas is a site Principal Investigator for a Vertex sponsored clinical trial on APOL1 mediated kidney disease. Shengliang He has no conflicts of interests to disclose.

## Data Availability

The data that support the findings of this study are available on request from the corresponding author.

## References

[1] OPTN Minority Affairs Committee , “Race and Hepatitis C Status no Longer Included in Calculation Used to Estimate Deceased Donor Kidney Function; Policy Change Anticipated to Increase Equity,” https://optn.transplant.hrsa.gov/news/race‐and‐hepatitis‐c‐status‐no‐longer‐included‐in‐calculation‐used‐to‐estimate‐deceased‐donor‐kidney‐function‐policy‐change‐anticipated‐to‐increase‐equity/.

[2] J. Miller , G. R. Lyden , W. T. McKinney , J. J. Snyder , and A. K. Israni , “Impacts of Removing Race From the Calculation of the Kidney Donor Profile Index,” American Journal of Transplantation 23, no. 5 (2023): 636–641, 10.1016/j.ajt.2022.12.016. 36695678

[3] D. S. Goldberg , P. L. Abt , and E. A. Blumberg , “Trial of Transplantation of HCV‐Infected Kidneys Into Uninfected Recipients,” New England Journal of Medicine 376, no. 24 (2017): 2394–2395, 10.1056/NEJMc1705221. 28459186

[4] C. M. Durand , M. G. Bowring , and D. M. Brown , “Direct‐Acting Antiviral Prophylaxis in Kidney Transplantation from Hepatitis C Virus–Infected Donors to Noninfected Recipients,” Annals of Internal Medicine 168, no. 8 (2018): 533–540, 10.7326/m17-2871. 29507971 PMC6108432

[5] Organ Procurement and Transplantation Network . The New Kidney Allocation System (Kas) Frequently Asked Questions. (2014) Retrieved November 10, 2023, from https://optn.transplant.hrsa.gov/professionals/by‐topic/guidance/thenew‐kidneyallocation‐system‐kas‐frequently‐asked‐questions/#bookmark5.

[6] P. S. Rao , D. E. Schaubel , and M. K. Guidinger , “A Comprehensive Risk Quantification Score for Deceased Donor Kidneys: The Kidney Donor Risk Index,” Transplantation 88, no. 2 (2009): 231–236, 10.1097/TP.0b013e3181ac620b. 19623019

[7] J. M. Miller , K. Poff , and J. N. Howell , “Updating the Kidney Donor Risk Index: Removing Donor Race and Hepatitis C Virus Status,” American Journal of Transplantation 25, no. 6 (2025): 1245–1252, 10.1016/j.ajt.2025.01.015. 39832693

[8] J. J. Friedewald , C. J. Samana , and B. L. Kasiske , “The Kidney Allocation System,” Surgical Clinics of North America 93, no. 6 (2013): 1395–1406, 10.1016/j.suc.2013.08.007. 24206858

[9] R. Daloul , T. E. Pesavento , D. S. Goldberg , and P. P. Reese , “A Review of Kidney Transplantation From HCV‐Viremic Donors Into HCV‐Negative Recipients,” Kidney International 100, no. 6 (2021): 1190–1198, 10.1016/j.kint.2021.06.034. 34237327

[10] L. Sibulesky , C. E. Kling , A. P. Limaye , and C. K. Johnson , “Is Kidney Donor Profile Index (KDPI) Valid for Hepatitis C Aviremic Kidneys?,” Annals of Transplantation 22 (2017): 663–664, 10.12659/aot.905428. 29104280 PMC6248315

[11] M. Yazawa , V. Balaraman , and M. Tsujita , “Donor Hepatitis C Antibody Positivity Misclassifies Kidney Donor Profile Index in Non‐Hepatitis C‐Infected Donors: Time to Revise the Kidney Donor Profile Index—a Retrospective Cohort Study,” Transplant International 33, no. 12 (2020): 1732–1744, 10.1111/tri.13743. 32935416

[12] S. He , C. P. Thomas , A. E. Gunderson , P. Ten Eyck , and A. I. Reed , “Outcomes of High Kidney Donor Profile Index Hepatitis C Nucleic Acid Testing Positive Kidneys are Equivalent to Matched Hepatitis C Nucleic Acid Testing Negative Kidneys,” Transplantation Direct 11, no. 7 (2025): e1827, 10.1097/txd.0000000000001827. 40590005 PMC12208634

[13] G. Guan , I. Ashlagi , and M. L. Melcher , “Transplant Surgeons Already Account for Inaccuracies in the Kidney Donor Profile Index (KDPI) Calculation,” Clinical Transplantation 38, no. 5 (2024): e15323, 10.1111/ctr.15323. 38690616

[14] R. E. Patzer , “Bridging Racial Disparities in Access to Kidney Transplantation in the United States,” Journal of the American Society of Nephrology 35, no. 7 (2024): 959–961, 10.1681/asn.0000000000000366. 38985123 PMC11230722

[15] P. N. Martins , M. N. Mustian , and P. A. Maclennan , “Impact of the new Kidney Allocation System A2/A2B → B Policy on Access to Transplantation Among Minority Candidates,” American Journal of Transplantation 18, no. 8 (2018): 1947–1953, 10.1111/ajt.14719. 29509285 PMC6105461

[16] Health Resources & Services Administration , “Refit Kidney Donor Profile Index Without Race and Hepatitis C​ Virus,” https://www.hrsa.gov/optn/policies‐bylaws/public‐comment/refit‐kidney‐donor‐profile‐index‐without‐race‐hepatitis‐c‐virus.

[17] T. M. Duello , S. Rivedal , C. Wickland , and A. Weller , “Race and Genetics Versus ‘Race’ in Genetics,” Evolution, Medicine, and Public Health 9, no. 1 (2021): 232–245, 10.1093/emph/eoab018. 34815885 PMC8604262

[18] W. C. Crannell , J. D. Perkins , N. Leca , and C. E. Kling , “Deceased Donor Kidneys are Discarded at Higher Rates When Labeled as High Kidney Donor Profile Index,” American Journal of Transplantation 22, no. 12 (2022): 3087–3092, 10.1111/ajt.17197. 36088649

[19] Health Resources and Services Administration | HRSA (.gov), “Allocation out of OPTN Sequence,” https://www.hrsa.gov/optn/policies‐bylaws/policy‐issues/allocation‐out‐of‐sequence‐aoos.

[20] J. T. Adler and S. Sharma , “Out‐of‐Sequence Allocation: A Necessary Innovation or a new Inequity in Transplantation?,” American Journal of Transplantation 25, no. 2 (2025): 234–236, 10.1016/j.ajt.2024.09.022. 39326851

[21] B. P. Concepcion , Y. El Kassis , and M. A. Josephson , “Is the Current Practice of out‐of‐Sequence Kidney Allocation Justified? Assessing Outcomes, Utility, Efficiency, and Equity,” Clinical Journal of the American Society of Nephrology (2026): 541–543, 10.2215/cjn.0000001043. 41817589 PMC13065198

[22] A. Attia , K. Sasaki , and A. J. Kwong , “Out‐of‐Sequence or Out of Options? Rethinking Liver Allocation in a Crisis of Confidence,” American Journal of Transplantation 26, no. 6 (2026): 1213–1214, 10.1016/j.ajt.2026.02.029. 41771356

[23] Health Resources and Services Administration | HRSA (.gov), “Strengthening the OPTN Through Data‐Driven Decision Making,” https://www.hrsa.gov/optn‐modernization/updates/august‐2025.

[24] K. Bradbrook , D. Klassen , A. B. Massie , and D. E. Stewart , “Does a Changing Donor Pool Explain the Recent Rise in the United States Kidney Nonuse Rate?,” American Journal of Transplantation 25, no. 8 (2025): 1685–1695, 10.1016/j.ajt.2025.02.004. 39947400

